# Receptor antibody time‐resolved A2 phospholipase bead immunochromatography and its application in idiopathic membranous nephropathy

**DOI:** 10.1002/jcla.23508

**Published:** 2020-08-06

**Authors:** Biao Huang, Xue Yang, Wenchen Zhang, Jian Wu, Pengfei Liu, Zhigang Hu, Tingting Wang

**Affiliations:** ^1^ College of Life Sciences and Medicine Zhejiang Sci‐Tech University Hangzhou China; ^2^ The Affiliated Wuxi Children's Hospital of Nanjing Medical University Wuxi People's Hospital affiliated to Nanjing Medical University Wuxi China; ^3^ The First People's Hospital of Yancheng City Yancheng China; ^4^ The Jiangyin Clinical College of Xuzhou Medical University Jiangyin China

**Keywords:** idiopathic membranous nephropathy, immunochromatography, PLA2R antibody, Time‐resolved fluorescent bead

## Abstract

**Objective:**

To detect phospholipase A2 receptor (PLA2R) antibody by established time‐resolved fluorescent bead immunochromatographic assay.

**Methods:**

The reaction time of coupling, pH of the reaction, and coupling ratio of the label to PLA2R were determined. The EDC method was used to covalently couple PLA2R to time‐resolved fluorescent beads, which were sprayed onto a bonding pad. PLA2R and rabbit anti‐PLA2R antibody sprayed onto a nitrocellulose membrane were used as detection and quality control lines, respectively. Immunochromatographic test strips were prepared to enable rapid detection of PLA2R antibodies. Various technical indicators were evaluated, and the correlation among this method, enzyme‐linked immunosorbent assay (ELISA), and serum analysis was examined.

**Results:**

The pH suitable for labeling was 6.5. The optimal mass ratio of PLA2R protein to fluorescent beads was 0.08:1, and the reaction time of coupling was at least 1.5 hours. The appropriate spray film size of the coupled fluorescent bead was 5 μL/cm, and the appropriate staining concentration of the test line was 0.28 mg/mL. Further, 80 µL of sample was required for the test, and the result was obtained in only 15 minutes. The measurable range of this method was 5‐1500 RU/mL. Intra‐ and inter‐assay coefficients of variation were 7.61% and 11.07%, respectively, with an average recovery rate of 93.77%. The method showed a good correlation with ELISA, with a correlation coefficient of 0.936.

**Conclusions:**

This method could better meet the clinical demand for idiopathic membranous nephropathy (IMN) detection.

## Graphical abstract

Eighty microliter of sample was required for the test, and the result was obtained in only 15 minutes. The measurable range of this method was 5‐1500 RU/mL. Intra‐ and inter‐assay coefficients of variation were 7.61% and 11.07%, respectively, with an average recovery rate of 93.77%. The method showed a good correlation with ELISA, with a correlation coefficient of .936.

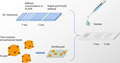

## 1 INTRODUCTION

Kidney disease occurs in 10.8% of Chinese adults. Nephrotic syndrome is a group of clinical syndromes involving increased glomerular basement membrane permeability and high levels of proteinuria, hypoproteinemia, edema, and hyperlipidemia. Among these diseases, membranous nephropathy (MN) is a common cause of adult nephrotic syndrome.^1^, ^2^

Membranous nephropathy is a group of diseases characterized by glomerular basement membrane epithelial cell immune complex deposition with diffuse thickening of the glomerular basement membrane. MN is often insidious. Approximately 20% of patients have asymptomatic, non‐renal disease proteinuria. Some patients realize their condition only after learning that they have proteinuria during routine physical examination. However, obtaining a proper diagnosis by renal puncture is difficult, and thus, the condition may be worsened before disease detection. MN can be divided into idiopathic membranous nephropathy (IMN) and secondary membranous nephropathy (SMN). IMN accounts for approximately two‐thirds of cases, whereas SMN accounts for approximately one‐third of cases and includes autoimmune diseases, infections, tumors, and drugs.^3^, ^4^ The characteristic pathological changes in IMN are the deposition of a large number of immune complexes on the glomerular capillary epithelial side. The clinical progress is slow, and some patients can spontaneously recover, but 30%‐40% of patients eventually develop final‐stage renal disease or die.^5^ IMN is mainly diagnosed by renal puncture and histological examination.^6^ However, this method has limitations such as a long inspection time, high trauma risk, and difficulty in carrying out the procedure in ordinary hospitals. Phospholipase A2 receptor (PLA2R) was first discovered in 2009 by Beck and others.^4^ This receptor is the target antigen of adult IMN and is detected by Western blotting. Antibodies for this receptor are observed in the serum of 70% of patients with IMN.^4^ A PLA2R‐lgG ELISA Kit was developed to simplify antibody detection that shows an IMN detection rate between 40% and 70%.^7^, ^8^, ^9^ However, the detection time is more than 2 hours and is not individualized. To overcome these limitations, we developed a time‐resolved fluorescent bead immunochromatographic technique to establish a rapid detection method for PLA2R antibody on an individual patient basis. We also evaluated various technical indicators and explored whether this method can be applied in clinical trials.

## 2 METHODS

### 2.1 Main materials and instruments

PLA2R antigen, rabbit anti‐PLA2R antibody, and the PLA2R‐IgG standard were provided by KeyGen Biotechnology Co., Ltd. Time‐resolved fluorescent nanospheres were purchased from Shanghai Bomai Biotechnology Co., Ltd. The absorbent paper, sample pad, bonding pad, nitrocellulose membrane (NC film), and card case were purchased from Shanghai Jieyi Biotechnology Co., Ltd. 1‐(3‐Dimethylaminopropyl)‐3‐ethylcarbodiimide hydrochloride (EDC) and *N*‐hydroxysuccinimide (NHS) were purchased from Sigma. The film meter was obtained from Shanghai Jieyi Biotechnology Co., Ltd. The fluorescent immunoassay analyzer was provided by KeyGen Biotechnology Co., Ltd. The enzyme‐linked immunosorbent assay (ELISA) kit for PLA2R‐IgG was from Euroimmun Co., Ltd. Other reagents were of analytical grade. Clinical serum specimens were obtained from Wuxi People's Hospital. All patients provided informed consent prior to study participation. The Wuxi People's Hospital Clinical New Technology and Research Ethics Committee approved this study (No.: kyl2016001).

### 2.2 Preparation of main reagents

#### 2.2.1 Preparation of pH 5.0, 0.1 mol/L MES solution

MES (2.132 g) was added to 100 mL of water and stirred to dissolve the MES completely; the pH was adjusted to 5.0 using 10 mol/L NaOH solution.

#### 2.2.2 Preparation of sealing solution

Bovine serum albumin (1.25 g) was dissolved in 25 mL of 0.01 mol/L phosphate buffer (PB, pH 7.5).

#### 2.2.3 Preparation of scribing buffer

Trehalose (0.2 g) was mixed with 0.6 mL of methanol and dissolved in 9.4 mL of 0.01 mol/L PB (pH 7.5).

#### 2.2.4 Preparation of suspension

Bovine serum albumin (1 g) and trehalose (5 g) were mixed. Next, 25 μL of Tween‐20 was added and this mixture was dissolved in 100 mL of 0.01 mol/L PB (pH 7.5). The solution was mixed by stirring.

### 2.3 Selection conditions for NC film coating

Different concentrations of PLA2R antigen were plated at 1.0 μL/cm onto the NC membrane as a detection line (T line). At the same time, the rabbit anti‐PLA2R antibody was also drawn at 1.0 μL/cm with a spectator and used as a quality control line and to judge whether the reaction is complete. The NC membrane was dried at room temperature for 12 hours.

### 2.4 Selection of PLA2R protein labeling conditions

Time‐resolved phospholipase beads were added to a centrifuge tube, an equal volume of pH 5.0, 0.1 mol/L MES buffer was added, and the solution was mixed. Ultrasonic cleaning was performed for 5 minutes, followed by centrifugation at 18894.2 *g* for 15 minutes. After discarding the supernatant, the pellet was resuspended in an equal volume of pH 5.0, 0.1 mol/L MES buffer. NHS was dissolved in 0.1 mol/L MES buffer. The cleaned fluorescent microspheres were added in the buffer, and the sample was shaken at room temperature for 30 minutes. After centrifugation at 18894.2 *g* for 30 minutes to remove the supernatant, the fluorescent microspheres were activated. EDC, NHS, and fluorescent microspheres were activated in a mass ratio of 2:1:2. Next, 0.1 mol/L MES buffer at different pH levels was added to resuspend the ultrasonic mixture and washed twice. The supernatant was discarded after centrifugation, and the washed microspheres were resuspended in an equal volume of 0.1 mol/L MES buffer and ultrasonically mixed. Different amounts of PLA2R protein were selected to react with 1 mg of activated fluorescent microspheres, which were mixed during addition. The reaction was carried out for 2 hours at room temperature. The labeled microspheres were centrifuged at 18894.2 *g* for 30 minutes to remove the supernatant. An equal volume of blocking solution was added to the microspheres. After resuspending the solution by ultrasound, the reaction was allowed to stand at room temperature for 1 hour and then centrifuged at 18894.2 *g* for 20 minutes. The supernatant was discarded, and an equal volume of the suspension was added and resuspended by ultrasound. After centrifugation at 18894.2 *g* for 20 minutes in a high‐speed refrigerated centrifuge, the supernatant was discarded. The final concentration of PLA2R protein‐conjugated fluorescent microspheres was 0.2 mg/mL after adding the resuspension. The film was sprayed with this solution.

### 2.5 Selection of spray film conditions

Different amounts of labeled fluorescent microspheres were sprayed onto the bonding pad. After drying, the test card was assembled with the lined NC film.

### 2.6 Assembly of fluorescent immunochromatographic test strips

In an environment of less than 35% humidity and stable temperature of 20‐25°C, the NC film with the T line and C line, and the bonding pad with labeled fluorescent microspheres were adhered to the polyvinyl chloride substrate. The sample pad and absorbent paper were attached to form a microfiltration system, which was cut to a width of 0.4 cm. The test strip was prepared and placed in the card case to form a strip. The aluminum film bag was sealed and stored.

### 2.7 Test method

A double‐antigen sandwich method was used to establish an assay for detecting PLA2R antibody; 80 µL of standard or test serum was added to the well and incubated at room temperature for 15 minutes. The test strip was inserted into the card insertion port of the fluorescence detecting device, and readings were obtained using the instrument. The fluorescence count was determined, and the corresponding concentration was obtained according to the set standard curve.

### 2.8 Methodological assessment

#### 2.8.1 Minimum detection limit

The minimum concentration that could be reliably identified from the background of the test was determined, and the average (x) and standard deviation (s) were obtained from 10 sets of standard curve zero tube counts. The value of (x + 2s) was calculated, and the corresponding concentration on the standard curve was detected.

#### 2.8.2 Specificity

To investigate the degree of cross‐reactivity between the antibody and test substance and measure the specificity of the test, we used high concentrations of hemoglobin, bilirubin, and triglyceride as samples.

#### 2.8.3 Recovery rate

A high‐concentration standard was added to a sample of known concentration, and the sample was measured according to the detection method. The ratio of the measured value to the theoretical value was calculated.

#### 2.8.4 Precision

The quality control of the low and high medium groups was tested 10 times, and the mean value, standard deviation, and coefficient of variation (CV) were calculated.

### 2.9 Comparison with PLA2R‐IgG ELISA kits

Thirty samples were simultaneously tested using ELISA Kits and time‐resolved fluorescent microsphere immunochromatographic test strips. Correlation analysis was performed between the two methods.

## 3 RESULTS

### 3.1 Selection of PLA2R protein labeling conditions

#### 3.1.1 Effect of pH on labeling

**Figure 1:**
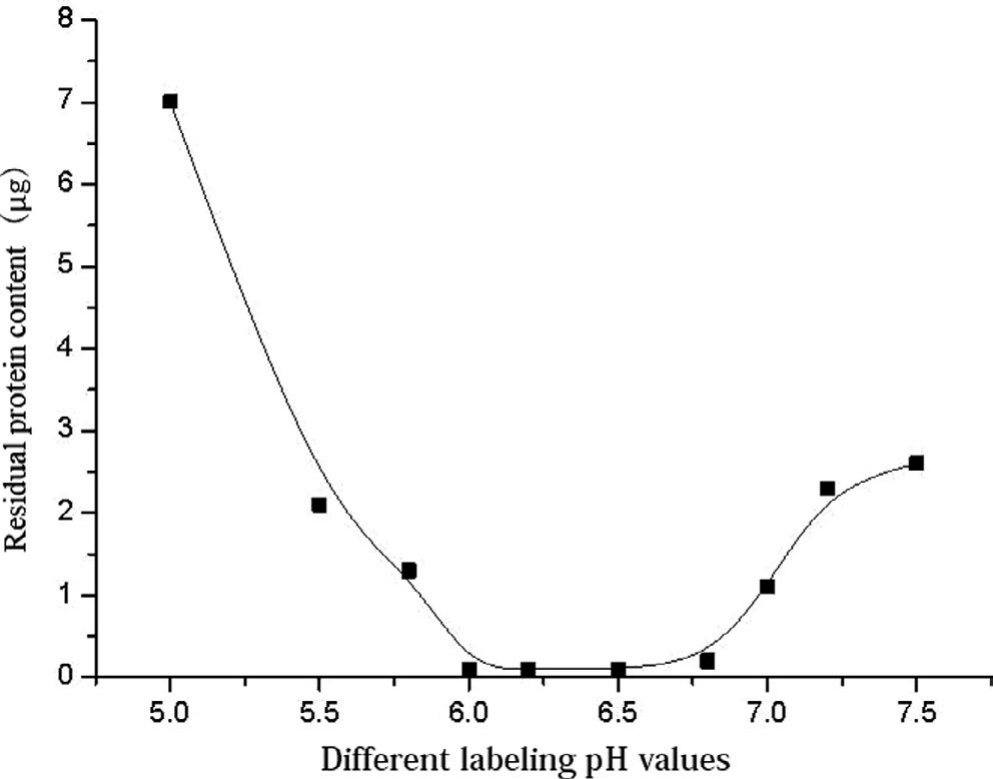
Residual protein concentration in the supernatant after labeling at different pH values

Labeling was performed using 0.1 mol/L MES buffer at pH 5.0, 5.5, 5.8, 6.0, 6.2, 6.5, 6.8, 7.0, 7.2, and 7.5, and 1‐μg microspheres were labeled with 80 μg protein and centrifuged after the reaction. The concentration of residual protein in the supernatant was determined by BCA protein assay (Figure 1). When the pH condition of labeling was between 6.0 and 6.8, the protein concentration in the supernatant was low, and most of the protein was labeled onto the beads. Thus, pH 6.5 was chosen as the optimum pH for labeling.

#### 3.1.2 The ratio of PLA2R protein to fluorescent beads during labeling

**Figure 2:**
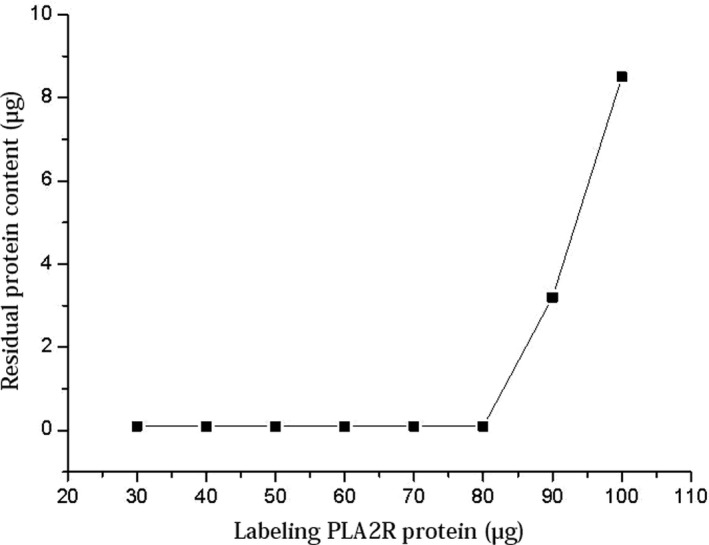
Amount of loaded and residual PLA2R protein during labeling

According to the labeling method, 1 mg of fluorescent beads was reacted with 30‐100 μg of PLA2R proteins at pH 6.5. After labeling, the residual protein concentration in the supernatant was determined by BCA protein assay. The results are shown in Figure 2.

As shown in Figure 2, when using 1 mg fluorescent beads and an amount of loaded PLA2R protein higher than 80 μg, the residual amount of protein in the supernatant began increasing, indicating that the protein level was excessive. Using PLA2R protein‐to‐beads ratio of 0.08:1 allowed labeling of as many proteins as possible on the fluorescent beads.

#### 3.1.3 Optimum duration for PLA2R protein labeling

**Figure 3:**
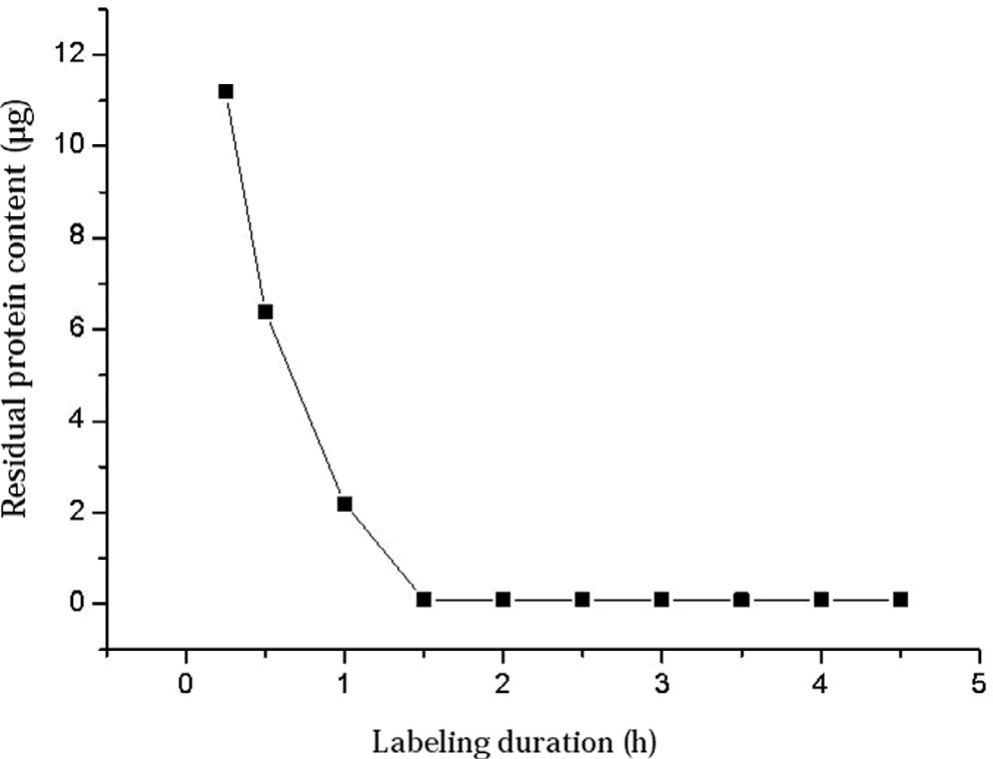
Relationship between labeling duration and amount of residual protein in the supernatant

According to the labeling method, at pH 6.5, 1 mg of beads and 80 μg of PLA2R protein were mixed and reacted for different durations following the standard steps, and the amount of residual protein in the supernatant was measured. As shown in Figure 3, when the labeling duration was more than 1.5 hours, the proteins were mostly labeled onto the beads. Thus, the minimum duration for labeling proteins onto the fluorescent beads was 1.5 hours.

#### 3.1.4 Selection of bead amount sprayed onto the membrane

**Figure 4:**
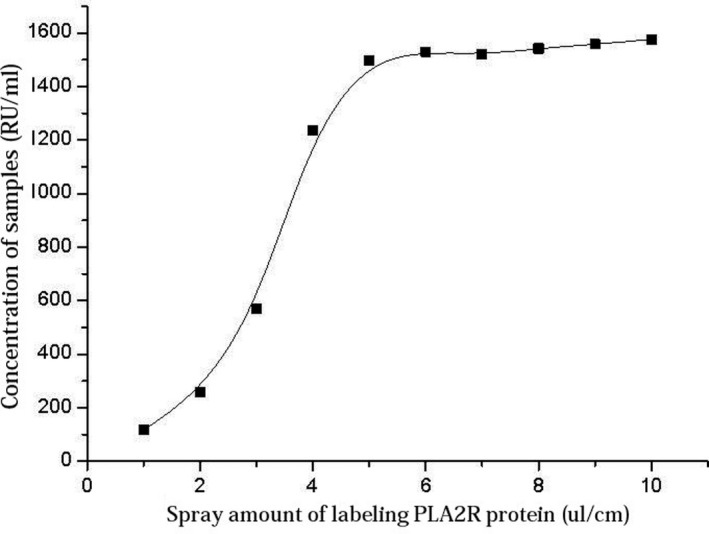
Relationship between spray amount of PLA2R protein labeling solution and the tested concentration of high‐concentration samples

The PLA2R protein‐conjugated fluorescent beads at a concentration of 0.2 mg/mL were sprayed onto the conjugate pad at concentrations of 1, 2, 3, 4, 5, 6, 7, 8, 9, and 10 μL/cm and dried with the marked NC film overnight. The testing strip was then assembled. First, highly concentrated serum was tested. As shown in Figure 4, when the amount of spray was less than 5 μL/cm, the amount of the labeled protein was insufficient compared with that of the serum. Thus, the amount of spray on the membrane should be higher than 5 μL/cm.

### 3.2 Concentration at T line

**Figure 5:**
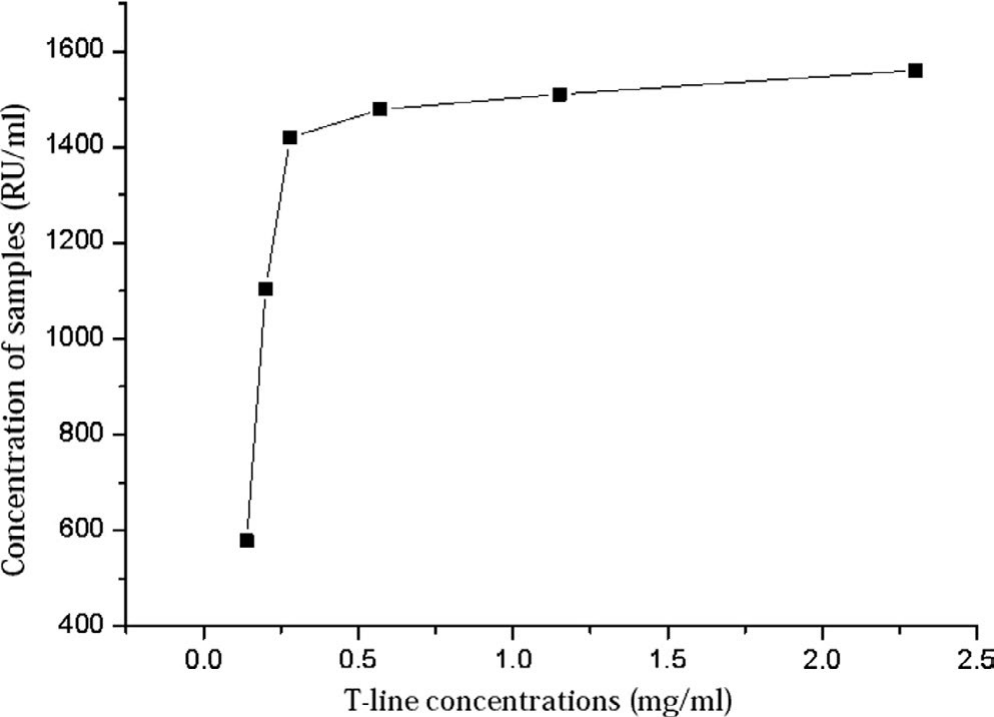
Results of high‐concentration samples at different T‐line concentrations

The PLA2R protein was diluted with capture antibody buffer solution at concentrations of 2.3, 1.15, 0.575, 0.28, 0.20, and 0.14 mg/mL, immobilized onto the nitrocellulose membrane with a dispenser at 1.0 μL/cm, and dried at room temperature for 12 hours. The C‐line antibody concentration was 1 mg/mL. Using the high‐concentration positive sample, the appropriate T‐line concentration was selected. When the T‐line concentration was below 0.28 mg/mL, the measured value of the high‐concentration sample increased with the T‐line concentration, as shown in Figure 5. When the concentration was higher than 0.28 mg/mL, the measured value of the high‐concentration sample was stable, indicating that the optimum concentration was 0.28 mg/mL.

### 3.3 Method evaluation

**Figure 6:**
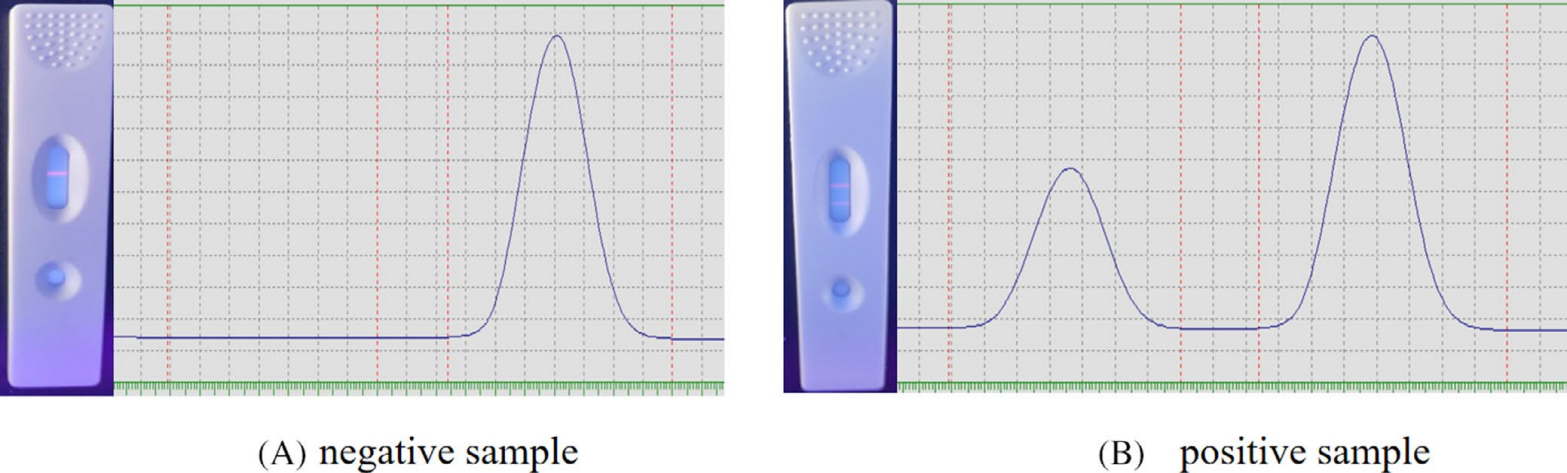
Images of the experiments detected by immunochromatographic strips

When the sample was added and reacted for 15 minutes under a UV lamp at a wavelength of 365 nm, the detection line (T) and quality control line (C) could be observed; the line close to the sample addition hole is the T line, and the peak pattern could be observed on a fluorescence immunoassay analyzer, and the corresponding fluorescence values could be obtained, as shown in Figure 6.

**Figure 7:**
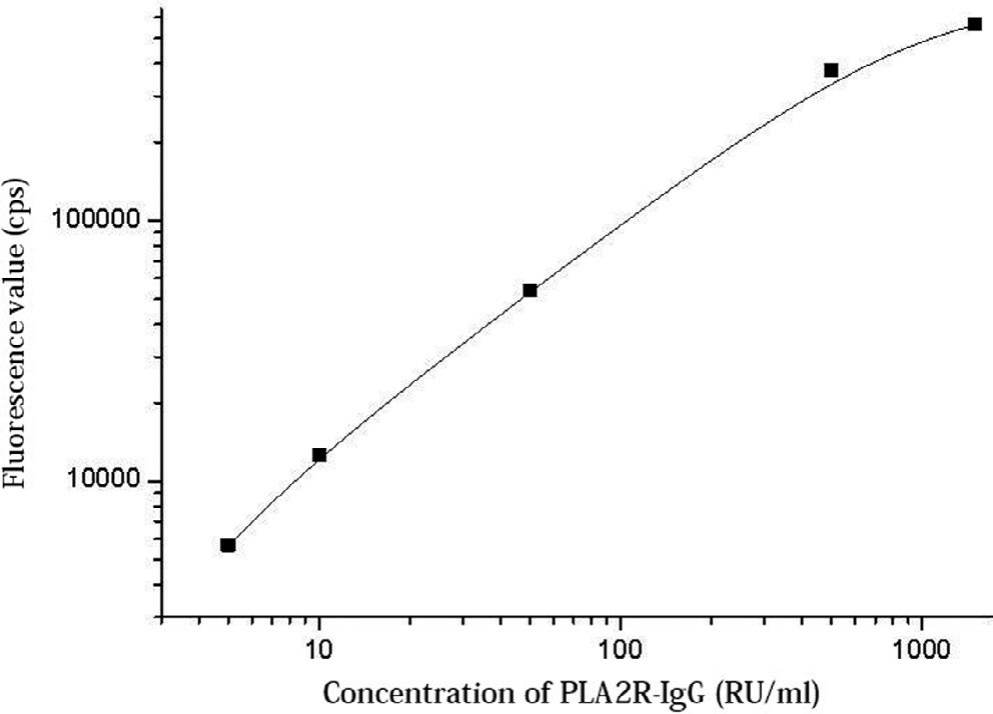
Standard curve of PLA2R‐IgG TRFIA‐POCT

Standard samples of different concentrations were tested to obtain a standard curve, as shown in Figure 7, over a measurement range of 5‐1500 RU/mL.

The technical indices of the method are as described in the following sections.

#### 3.3.1 Sensitivity

The average value (x) and standard deviation (s) were calculated from 10 sets of standard curve zero counts, and $\bar{x}+2s$ was calculated. The corresponding concentration on the standard curve was considered as the sensitivity and showed a value of 0.6 RU/mL.

#### 3.3.2 Specificity

Samples of 3 mg/mL hemoglobin, 2 mg/mL bilirubin, and 10 mg/mL triglyceride were tested, and the measured concentrations were all lower than 0.6 RU/mL, indicating that these substances did not affect the reaction. Thus, the reaction was highly specific.

#### 3.3.3 Recovery rate

A high‐concentration standard sample was added to a known low‐concentration sample (1:9, V/V), and the sample was tested. The ratio of the measured value to the theoretical value was calculated, and the average recovery rate was 93.77%, as shown in Table 1.

**Table 1:** Recovery experiment

Low concentration of anti‐PLA2R (RU/mL)	Concentration of sample added with anti‐PLA2R (RU/mL)	Measured value (RU/mL)	Predicated value (RU/mL)	Recovery rate%
7.61	1500	149.52	156.85	95.33%
53.71	1500	182.89	198.34	92.21%
Average recovery				93.77%

#### 3.3.4 Precision rate

The low, medium, and high‐concentration quality control groups were tested 10 times, with 3 sets of reagents. The intra‐assay CV was 7.61%, and the inter‐assay CV was 11.07%.

#### 3.3.5 Comparison with PLA2R‐IgG ELISA Kit

**Figure 8:**
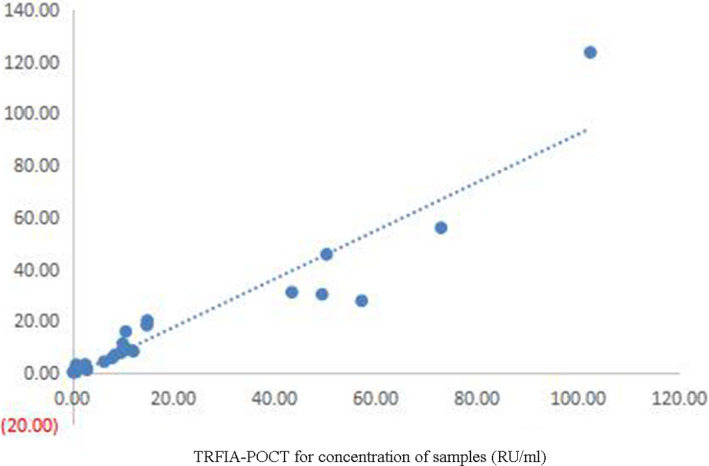
Comparison of different methods for detecting serum concentrations

Thirty samples were simultaneously tested using an ELISA Kit and time‐resolved fluorescent immunochromatography test strips, and 7 samples with concentrations lower than the minimum sensitivity were excluded. Correlation analysis was performed between the two methods. As shown in Figure 8, the linear correlation between the two methods was y = 0.9302x − 1.0105 and *R* ^2^ = .8773. At a cutoff value of 14 RU/mL, the positive and negative results given by the two methods exhibited 100% agreement, as shown in Table 2.

**Table 2:** Correspondence rate of positive and negative results of serum measured by different methods

TRFIA‐POCT	ELISA	Negative	Positive
Negative		19	0
Positive		0	11

## 4 DISCUSSION

PLA2R autoantibodies are an ideal marker for diagnosing MN and determining its activity, treatment timing, drug selection, and efficacy. Western blotting was first used to detect PLA2R autoantibodies^4^ but showed limited sensitivity and is mainly used for qualitative or semi‐quantitative analysis. The protocol is complicated, and the amount of sample that can be evaluated in each test is limited, making this method unsuitable for large‐scale testing. The detection rate of IMN using PLA2R‐IgG ELISA reagents is between 40% and 70%.^7^, ^8^, ^9^ Although the protocol is less complicated than Western blotting, the detection rate is lower. In recent years, immunoassay methods have been developed rapidly, among which dissociation‐enhanced time‐resolved fluoroimmunoassay (TRFIA) can amplify the fluorescence signal by one‐million‐fold, and thus is more sensitive than both Western blotting and ELISA. The measurement range is also more comprehensive. The detection rate of IMN is 89%, which is much higher than that of the PLA2R‐IgG ELISA reagent.^10^, ^11^ However, the ELISA or TRFIA reactions require more than 2 hours, and they cannot test samples from a single person, and thus do not meet the clinical demand. Although traditional colloidal gold immunochromatography can rapidly test samples from a single patient, its sensitivity is low, and it is generally used for qualitative detection. To further improve the sensitivity of detection and perform quantitative analysis in a convenient and rapid manner based on immunochromatography and develop a time‐resolved fluorescent immunochromatography method to detect PLA2R antibodies, we combined time‐resolved fluoroimmunoassay, fluorescent beads, and immunochromatography. The fluorescent beads are solid microparticles loaded with fluorescence molecules with diameters from nanometers to micrometers (0.01‐10 μm) and can be excited by external energy stimulation and emit fluorescence signals. Because of its relatively stable morphological structure, luminescence behavior, and stable and robust emission, fluorescent immunochromatography shows good potential for clinical application.^12^ Time‐resolved fluorescent immunochromatography uses fluorescent nanoparticles coated with lanthanide (Eu) chelate as a marker, combined with the long decay fluorescence and signal amplification effects. It shows high sensitivity, high stability, excellent specificity, a wide linear range, and a short detection time.^13^ In this study, nano‐fluorescent beads coated with the rare earth ion EU were used as markers rather than traditional colloidal gold particles. EU not only has a strong fluorescence signal under ultraviolet excitation but also has a long decay time. Because the NC membrane also exhibits a fluorescent signal under ultraviolet excitation, we detected the time delay after excitation. We only measured the fluorescent signal of rare earth ions after the background fluorescence had disappeared, eliminating background interference and improving detection sensitivity. Previously, PLA2R antibody was detected by a two‐step method.^10^, ^11^, ^14^, ^15^ To make the reaction more convenient and rapid, we used the double‐antigen sandwich method to achieve a one‐step method. By determining and optimizing the conditions such as labeling time, reaction pH, and amount of spray, we developed PLA2R TRFIA immunochromatography. The time‐resolved fluorescence technology showed a detection sensitivity of 0.6 RU/ML, wide measurement range, and excellent stability, and the technical indices meet the requirements for clinical examination. The serum test is consistent with the clinical results of ELISA; the application of the immunochromatography method makes the test faster and simpler. Only 80 µL of serum is required, and the result is obtained in only 15 minutes, which meets the clinical demand for better IMN detection.

## AUTHOR CONTRIBUTIONS

BH, XY, and WZ conducted the detections and drafted the article. WJ and LP collected the data. ZH and T.W designed the study, purchased reagents, and revised the article. All the authors approved the submission.
